# *Bacillus amyloliquefaciens* and *Saccharomyces cerevisiae* feed supplements improve growth performance and gut mucosal architecture with modulations on cecal microbiota in red-feathered native chickens

**DOI:** 10.5713/ab.21.0318

**Published:** 2022-01-03

**Authors:** Tzu-Tai Lee, Chung-Hsi Chou, Chinling Wang, Hsuan-Ying Lu, Wen-Yuan Yang

**Affiliations:** 1 Department of Animal Science, National Chung Hsing University, Taichung, 402, Taiwan; 2 The iEGG and Animal Biotechnology Center, National Chung Hsing University, Taichung, 402, Taiwan; 3 Department of Veterinary Medicine, School of Veterinary Medicine, National Taiwan University, Taipei City 106, Taiwan; 4 Zoonoses Research Center and School of Veterinary Medicine, National Taiwan University, Taipei City, 106, Taiwan; 5 Department of Basic Sciences, College of Veterinary Medicine, Mississippi State University, P.O. Box 6100, Mississippi State, MS 39762, USA

**Keywords:** *Bacillus amyloliquefaciens*, Growth Performance, Metagenomics, Microbiota Modulation, *Saccharomyces cerevisiae*

## Abstract

**Objective:**

The aim of study was to investigate the effects of in-feed supplementation of *Bacillus amyloliquefaciens* (BA) and *Saccharomyces cerevisiae* (SC) on growth performance, gut integrity, and microbiota modulations in red-feathered native chickens (RFCs).

**Methods:**

A total of 18,000 RFCs in a commercial farm were evenly assigned into two dietary treatments (control diet; 0.05% BA and 0.05% SC) by randomization and raised for 11 weeks in two separate houses. Fifty RFCs in each group were randomly selected and raised in the original house with the partition for performance evaluations at the age of 9 and 11 weeks. Six non-partitioned RFCs per group were randomly selected for analyses of intestinal architecture and 16S rRNA metagenomics.

**Results:**

Feeding BA and SC increased the body weight and body weight gain, significantly at the age of 11 weeks (p<0.05). The villus height/crypt ratio in the small intestines and Firmicutes to Bacteroidetes ratio were also notably increased (p<0.05). The supplementation did not disturb the microbial community structure but promote the featured microbial shifts characterized by the significant increments of *Bernesiella*, *Prevotellaceae_NK3B31_group*, and *Butyrucimonas*, following remarkable decrements of *Bacteroides*, *Rikenellaceae_RC9_gut_group*, and *Succinatimonas* in RFCs with growth benefits. Besides, functional pathways of peptidoglycan biosynthesis, nucleotide excision repair, glycolysis/gluconeogenesis, and aminoacyl transfer ribonucleic acid (tRNA) biosynthesis were significantly promoted (p< 0.05).

**Conclusion:**

In-feed supplementation of BA and SC enhanced the growth performance, improved mucosal architectures in small intestines, and modulated the cecal microbiota and metabolic pathways in RFCs.

## INTRODUCTION

Native chickens dominate the meat-type chicken market in Taiwan and Asian countries based on the culture of consumption and demands for traditional tastes. A popular breed known as red-feathered native chicken (RFC) is adopted to increase the resistance of heat stress and the desirable efficiency on body weight gain (BWG) when compared to other native chickens [1]. However, the long raising period of 12 to 14 weeks lowers the competitiveness in the market when compared to commercial broilers. Therefore, the industry seeks methodologies to improve the growth performance and feed efficiency of RFCs for competitive advantages in the market.

The health status of the gut determined the feed intake and the efficient assimilation of nutrients, thereafter, influencing the growth performance and poultry production. It is evident that the gut microbiota plays a crucial role in the maintenance of gut health, immune modulation, and disease development in chickens [2]. Based on the concerns about antimicrobial resistance, the use of antibiotic growth promoters (AGPs) for sustaining gut health in poultry has been prohibited, promoting the emerge of enteric diseases. Various alternatives to AGPs, such as direct-fed microbial (DFM), were studied and demonstrated to improve the overall gut health and growth performance in chickens [3,4].

Due to the health-promoting benefits and the ability to survive in harsh environmental conditions, the use of *Bacillus* as an AGP alternative has grown in popularity in recent years. Several studies showed that *Bacillus amyloliquefaciens* (BA) enhanced the digestion and absorption efficiency in the gut through secreted extracellular enzymes, including α-amylases, cellulase, metalloproteases, and proteases to promote gut health and growth performance [3,5]. Furthermore, the use of BA showed some modulations on cecal microbiota [5,6] and partial alleviation effects on compromised growth performance [7], indicating that the growth benefits may result from the interactions of gut microbiota in the host. Nonetheless, different species or strains of BA have shown different results, leading to inconsistent results on broiler growth performance [8,9].

*Saccharomyces cerevisiae* (SC) is a type of yeast extensively used in diets to improve health and productivity for various animal species. It was documented to confer beneficial effects on metabolic processes of digestion and nutrient utilization, health status, and the meat quality in broilers [10]. Besides, SC that carries mannanoligosaccharide (MOS) in the cell wall as a natural prebiotic that promotes the growth of beneficial microflora and inhibits the multiplication of gut pathogens [11]. The MOS has been shown to enhance feed efficiency on growth performance in poultry, including body weight (BW), feed intake, and feed conversion rate (FCR) [12].

Animal metabolism involves the interactions of different pathways that are modulated by the host’s gut microbiota [6]. They are regulated through microbial interactions and a range of metabolites produced by the microbial community members or the transformations from host molecules or diets [13]. Although different gut segments contribute to designated functions in the host, the ceca harbor the most diverse microbial communities for energy harvest and nutrient digestion, affecting gut health and growth performance of chickens [2,14]. Small intestines and ceca play crucial roles in nutrient absorption and energy harvest. On the other side, different probiotic species or strains that act on distinct sites, provide various modes of action, or exert varying levels of protective efficiency can generate synergistic effects [15]. Therefore, the utilization of multi-species or multi-strains probiotics can be beneficial for field applications. The purpose of this study was to evaluate the effects of BA and SC on growth performance, expecting to shorten the raising period in the field practice, and the correlation of the performance with the small intestinal improvement and cecal microbiota modulation at the field level, elucidating the mechanisms of this mixture to manipulate the intestinal environment in RFCs.

## MATERIALS AND METHODS

### Animal care

The procedure and animals used in this experiment were approved by the Animal Care and Use Committee of National Chung Hsing University, Taiwan (IACUC No. 107-014).

### Experimental design and diets

A total of 18,000 one-day-old male and female RFCs from commercial hatcheries were randomly and evenly assigned into two groups: experimental and control groups and placed in two separate houses with nets to avoid contact with wild birds. Afterward, fifty RFCs in each group were randomly selected and raised in the original house with the partition. Since RFCs were native chicken with a raising period of 12 to 14 weeks, the comparisons of performance were conducted at the time near marketing, setting to the age of 9 and 11 weeks. The end of the experiment was set to the time that RFCs reached the average BW of 2.4 kg required for marketing. All RFCs were raised in floor pens with wood shavings as litter and vaccinated against Infectious bronchitis, Newcastle disease, Chicken pox, Infectious bursal disease, Infectious coryza, and Infectious laryngotracheitis. The RFCs within the control group were fed a formulated corn-soybean-based (control) diet with no addiction, whereas the same control diets supplementing with the mixture of 0.05% BA and 0.05% SC were applied in the experimental group. The water and feed were provided *ad libitum* throughout the entire experiment. From week 1 to 3, weeks 3 to 6, weeks 7 to the end of the experiment, RFCs received the starter diets, grower diets, and finisher diets, respectively. Ingredients and nutrient levels of the control diet are described in Table 1.

### Strains and feed preparation

The BA Y2 and SC C1 strains were selected and applied as feed additives. They were kindly provided by Prof. Tzu-Tai Lee at National Chung Hsing University, isolated from feeds and wine grains, respectively. The BA Y2 and SC C1 were reactivated on lysogeny broth (LB) agar and yeast-mald (YM) agar at 37°C for 48 hours, respectively. Single colonies of BA Y2 and SC C1 were collected and inoculated into 10 mL LB and 10 mL YM and then incubated at 37°C for 24 hours. The cultured broth of BA Y2 or SC C1 was added into 90 mL LB or YM fresh broth for another cultivation of 24 hours at 37°C. Afterward, large-scaling culture through liquid fermentation was performed by the company at 37°C with 160 rpm for 24 hours. Powders of BA Y2 and SC C1 strains with a concentration of 10^9^ colony-forming unit (CFU)/g were obtained after freeze-drying. The BA Y2 and SC C1 were then mixed with a ratio of 1:1. The 0.5 kg of BA power with a concentration of 10^9^ CFU/g was mixed with 0.5 kg of SC powder with a concentration of 10^9^ CFU/g. One kilogram of the mixture was added into one ton of the control diet to form the experimental diet with 0.05% BA (5×10^6^ CFU/kg feed) and 0.05% SC (5×10^6^ CFU/kg feed).

### Evaluation of growth performance and intestinal villi morphology

The parameters of BW and BWG were applied to evaluate the growth performance by supplementation of BA and SC. The villus height, crypt depth villus, and villus height/crypt depth ratio (V/C ratio) in small intestines were measured to assess intestinal health for nutrient absorption. An increment of the V/C ratio was considered as an improvement of nutrient absorption. The BW and BWG were calculated from 50 RFCs within the partition per group at the times of 9 and 11 weeks, respectively. The FCR was calculated from the total amount of feed consumption by the total weight gain from all RFCs in each group. For histological evaluation of morphology in small intestines, six RFCs per group were randomly selected from non-partitioned RFCs in the house and euthanized at the age of 9 and 11 weeks. Jejunal and ileal segments were removed from the midway between the entry of the bile ducts and Meckel’s diverticulum and 10 cm proximal to the ileocecal junction. Jejunal and ileal segments with a length of approximately 2 to 3 cm were collected into 10% sodium phosphate-buffered formalin. The samples were cross-sectioned at 4 mm intervals and processed to paraffin-embedded blocks. The segments were cut at 4 to 5 μm and stained with hematoxylin and eosin for histological examinations. The measurements of villus height were conducted under the optical microscope. The length of the villus from the tip to the villus-crypt junction was recorded using Motic Image Plus 2.0 software (Microscope World, Carlsbad, CA, USA). The depth of the invagination between adjacent villi was determined as crypt depth. Twenty well-oriented and intact crypt-villus units from an intestinal cross-section of one chicken were selected. The average values of villus heights and crypt depths were calculated for the V/C ratio.

### 16S rRNA pyrosequencing

Six RFCs per group were randomly selected and euthanized from non-partitioned RFCs in the house by CO_2_ at the age of 4, 7, 9, and 11 weeks, respectively, to collect cecal contents through manual extrusion for 16S rRNA metagenomics analysis. The cecal contents from the control group were designated as W4.CC, W7.CC, W9.CC, and W11.CC based on the age of collections. The W4.PC, W7.PC, W9.PC, and W11.PC represented cecal contents from the experimental group collected at the age of 4, 7, 9, and 11 weeks. Cecal contents were placed in dry ice immediately when they were collected from chickens. Total genomic DNA was isolated from 150 mg of cecal contents using a Quick-DNA Fecal/Soil Microbe Miniprep Kit (Zymo Research, Irvine, CA, USA) following the manufacturer’s instructions. The sequencing of 16S rRNA was performed using HiSeq 2500 platform (Illumina, San Diego, CA, USA). The variable V3–V4 region of the 16S rRNA gene was PCR-amplified in a 25 Ml reaction mixture containing 12.5 μL 2X KAPA HiFi HotStart ReadyMix (Roche, Pleasanton, CA, USA), 0.75 μL of each 10 μm Illumina primer (forward primer-5′CCTA CGGGNGGCWGCAG 3′ and reverse primer-5′ GACTA CHVGGGTATCTAATCC 3′) with standard adapter sequences, and 1 μL of DNA template. The polymerase chain reaction (PCR) conditions were as follows: initial denaturation at 95°C for 3 minutes, 25 cycles of 95°C for 30 seconds, 55°C for 30 seconds, and 72°C for 30 seconds, and then final extension at 72°C for 5 minutes. After the clean-up of the amplicons, the index PCR was conducted by using a Nextera XT Index Kit (Illumina, San Diego, CA, USA) to attach a unique 8-bp barcode sequence to the adapters. The PCR products were purified and measured for the size and concentration by a Qsep100TM capillary electrophoresis system (BiOptic Inc., New Taipei City, Taiwan) and a Qubit 4.0 Fluorometer with Qubit dsDNA HS Assay Kit (Fisher Scientific, Waltham, MA, USA). The libraries were normalized, pooled to one tube with a final concentration of 10 pM, and sequenced on a MiSeq System using Illumina MiSeq Reagent Kit v3 (2×300 bp paired-end run).

### Data processing and statistical analysis

Paired-end sequences were merged through fast length adjustment of short reads (FLASH) v1.2.11 to obtain raw tags. Raw tags were de-multiplexed and filtered by Quantitative Insights into Microbial Ecology (Qiime) software v1.9.1 with the default quality criteria and a threshold Phred quality score of Q≥20 to have clean tags. Effective tags were obtained by filtering out and checking chimeric sequences through the UCHIME algorithm and Gold database. The operational taxonomic units (OTUs) were classified from effective tags by the 97% similarity of sequences using the UPARSE algorithm in USEARCH. The taxonomy-based analysis was conducted by RDP Classifier v2.11 with a cut-off of 80%, determining the matched taxon in the Silva v132 database. The diversities and statistical analyses for taxonomic profile differences were performed by Qiime v1.9.1, R v.3.3.1 (http://www.R-project.org/) with metagenomeSeq package, and Statistical Analysis Metagenomic Profiles (STAMP) software v2.1.3 with Welch’s t-test. The p-value was set to 0.05 for statistical significance. An algorithm for high-dimensional class comparisons between biological conditions, linear discriminant analysis effect size (LEfSe; http://huttenhower.sph.harvard.edu/lefse/), was conducted to determine the significant feature taxa between groups with linear discriminant analysis (LDA) scores of 3.5. Tax4Fun package in R v.3.3.1 was performed to blast the unigenes against the Kyoto encyclopedia of genes and genomes (KEGG) database for the prediction of functional pathways. The annotation information of KEGG Orthology (KO) from the KEGG database was acquired based on the relative abundance profile. The differential function pathway between BA+SC and the control group was determined at level 3.

Significant differences in BW, BWG, villus height, crypt depth, and V/C ratio between the control and experimental groups were determined by using SAS software version 9.4 (SAS Institute, Inc., Cary, NC, USA). Data of 16S rRNA metagenomics was subjected to R for analysis. Statements of statistical significance were based on the level of p<0.05.

## RESULTS

### Beneficial effects of BA and SC on growth performance

The effects of supplementing BA Y2 and SC C1 strains in feed on growth performance are shown in Table 2. The BW and BWG were significantly increased (p<0.05) in RFCs fed with BA and SC at the age of 11 weeks, facilitating these chickens to reach the BW required for marketing (2.4 kg). The lower value of overall FCR in the experimental group when compared to the control group was noted at the age of 9 and 11 weeks.

### Feeding BA and SC improved mucosal architectures in small intestines

**Figure 1:**
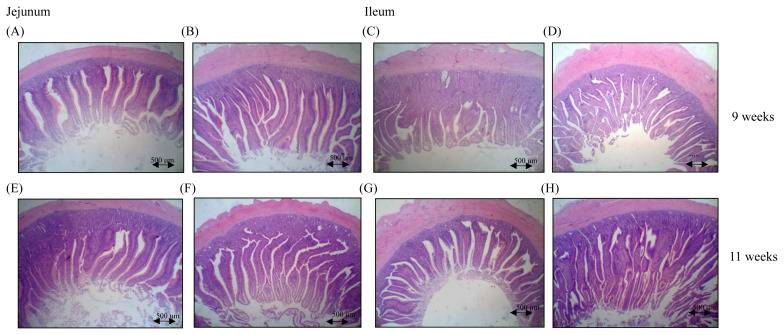
Effects of BA and SC on intestinal villi morphology. Six red-feathered native chickens (RFCs) per group were randomly selected and euthanized. Villus heights and crypt depths in the jejunum (A, B, E, F) and ileum (C, D, G, H) between the control and experimental groups were evaluated by hematoxylin and eosin (H&E) staining at the age of 9 weeks (A, B, C, D) and 11 weeks (E, F, G, H). Jejunal and ileal mucosa from chickens fed with BA and SC at the age of 9 weeks and 11 weeks both had longer villi than those observed in the control group. BA, *Bacillus amyloliquefaciens*; SC, *Saccharomyces cerevisiae*.

Histological examinations of jejunal and ileal mucosa demonstrated that chickens fed with BA Y2 and SC C1 at the age of 9 weeks and 11 weeks had longer villi than those observed in the control group (Figure 1). The numerical assessments of villus height, crypt depth, and V/C ratio are summarized in Table 3. The supplementation of BA Y2 and SC C1 increased villus heights, shortened crypt depths, or increased V/C ratio in the jejunum and/or ileum at the age of 9 and 11 weeks. In the jejunum, the V/C ratio was significantly increased at the age of 11 weeks (p<0.05) and the villus heights were significantly increased at the age of 9 weeks (p<0.05). For the ileum, the supplementation significantly increased villus heights (p<0.05) and V/C ratio (p<0.01) at the age of 11 weeks and decreased crypt depths at the age of 11 weeks (p<0.05). The mucosal architectures in the jejunum and ileum were significantly improved after the supplementation, particularly at the age of 11 weeks, showing the beneficial effects on nutrient absorption.

### Modulation effects on cecal microbiota by BA and SC supplements

**Figure 2:**
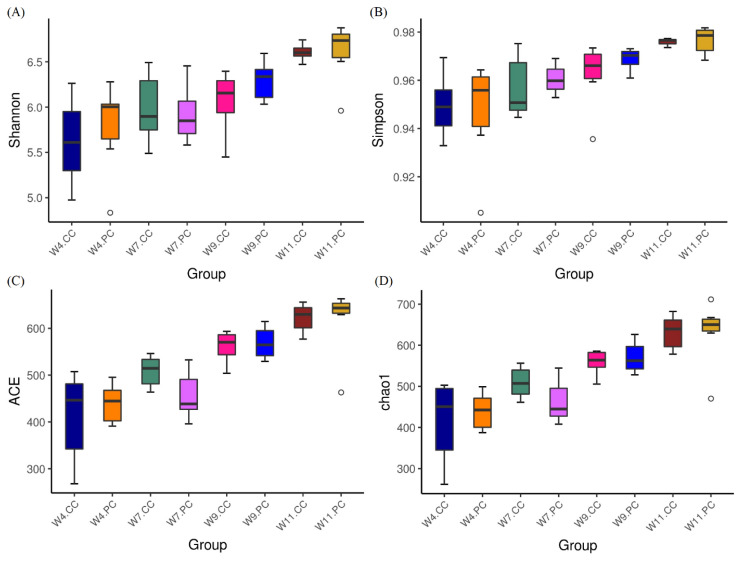
Analysis of alpha diversity in cecal microbiota. (A) Shannon index, (B) Simpson index, (C) abundance-based coverage estimator (ACE) index, and (D) Chao1 index. Results were shown as boxplots (n = 6 per group). Each box represents the interquartile range (IQR) between the first (25th percentiles) and third quartiles (75th percentiles) with the horizontal line that indicated the median. Species richness and diversity in the cecum increased with age in chickens fed with the control diets. The supplementation of BA and SC exerted varying effects on alpha-diversity in the cecum and promoted more species richness and diversity at the age of 11 weeks without a statistical significance. BA, *Bacillus amyloliquefaciens*; SC, *Saccharomyces cerevisiae*.

**Figure 3:**
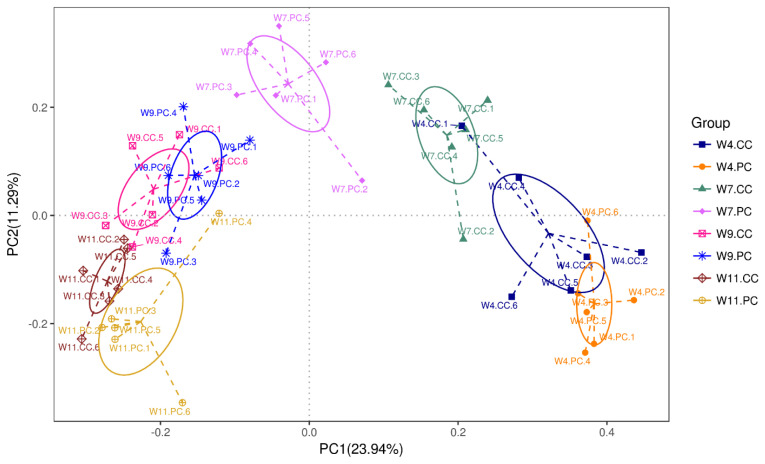
Beta-diversity of cecal microbiota between groups. Bray-Curtis principal coordinate analysis (PCoA) was conducted for evaluating the compositions and similarities of cecal microbiota of red-feathered native chickens (RFCs) between the control (CC) and experimental (PC) groups at the age of 4, 7, 9, and 11 weeks (n = 6 per group). A significant difference in cecal community profiles between the control and experimental groups was noted at the age of 7 weeks.

The analysis of alpha-diversity by Shannon, Simpson, abundance-based coverage estimator (ACE), and Chao1 indices (Figure 2) showed that the species richness and diversity in the cecum increased with age in chickens fed with the control diets. The supplementation of BA and SC exerted varying effects on alpha-diversity in the cecum but promoted more species richness and diversity at the age of 11 weeks. However, no significant difference was observed between the control and experimental groups at the age of 4, 7, 9, and 11 weeks. The results of beta diversity demonstrated that the principal components, PC1 and PC2, of the experimental group at the age of 7 weeks was distinctly separated with 23.94% and 11.29% variation, representing a significant difference in cecal community profiles between the control and experimental groups (Figure 3). No separation was noted between groups at the age of 4 and 9 weeks.

**Figure 4:**
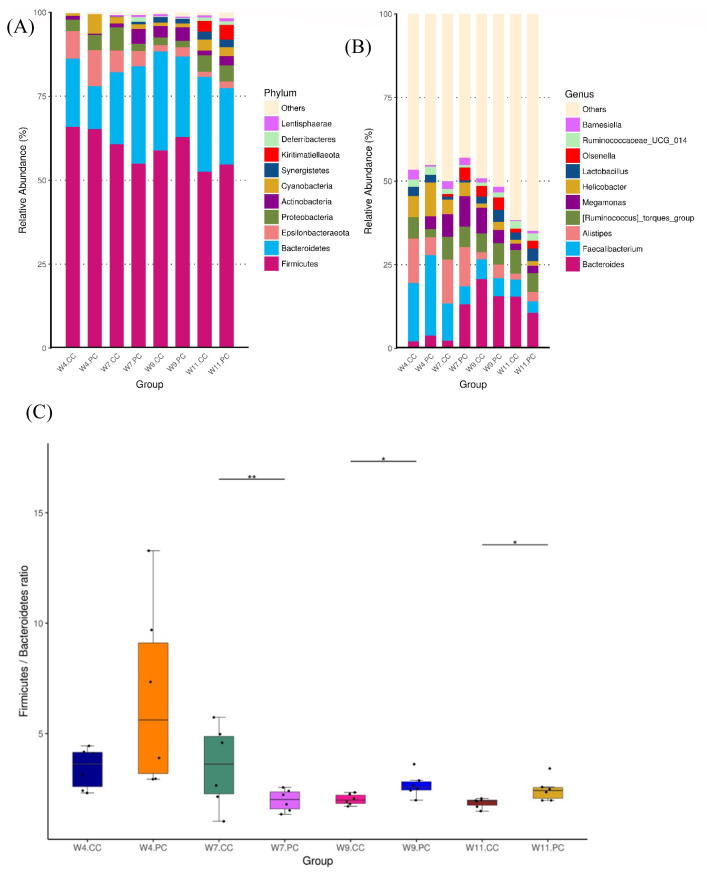
Analysis of microbial composition in cecal microbiota between groups (n = 6 per group). The average relative abundance of the bacterial taxon was presented as the bar within a group. The top 10 abundant taxa are shown at the level of phylum (A) and genus (B), respectively. The composition changed with age and *Bacteroides* converted into the dominant genus at the age of 9 weeks. The supplementation of BA and SC did not significantly alter microbial community structure at different ages of time (Figure 4B). However, the treatment significantly increased the cecal F/B ratio at the age of 9 and 11 weeks (4C). BA, *Bacillus amyloliquefaciens*; SC, *Saccharomyces cerevisiae*. Significance was detected by Kruskal-Wallis test: * p<0.05 and ** p<0.01.

**Figure 5:**
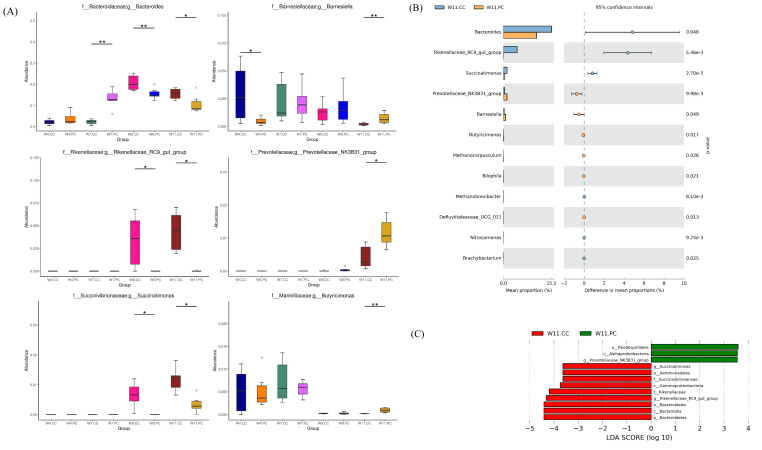
Identifications of the differential abundance of genera in cecal microbiota (n = 6 per group). (A) Analysis by metagenomeSeq: the differential abundance of microbes in the cecum between groups was analyzed at the age of 4, 7, 9, and 11 weeks: * p<0.05 and ** p<0.01. (B) STAMP analysis at same different ages of time. The bar plot demonstrated the differential abundance of genera between the control and experimental groups at the age of 11 weeks. Only significant features with p<0.05 (Welch’s t-test) were shown. Significant increments of *Bernesiella*, *Prevotellaceae_NK3B31_group*, and *Butyrucimonas* (p<0.05) were noted with remarkable decrements of *Bacteroides*, *Rikenellaceae_RC9_gut_group*, and *Succinatimonas* (p<0.05) (C) linear discriminant analysis effect size (LEfSe) analysis with linear discriminant analysis (LDA) score >3.5 at the age of 11 weeks. *Prevotellaceae_NK3B31_group*, *Rikenellaceae_RC9_gut_group*, and *Succinatimonas* were identified as differentially significant and biological feature taxa between groups (p<0.05). Identifications of the differential abundance of genera in cecal microbiota (n = 6 per group). (A) Analysis by metagenomeSeq: the differential abundance of microbes in the cecum between groups was analyzed at the age of 4, 7, 9, and 11 weeks: * p<0.05 and ** p<0.01. (B) STAMP analysis at same different ages of time. The bar plot demonstrated the differential abundance of genera between the control and experimental groups at the age of 11 weeks. Only significant features with p<0.05 (Welch’s t-test) were shown. Significant increments of *Bernesiella*, *Prevotellaceae_NK3B31_group*, and *Butyrucimonas* (p<0.05) were noted with remarkable decrements of *Bacteroides*, *Rikenellaceae_RC9_gut_group*, and *Succinatimonas* (p<0.05) (C) linear discriminant analysis effect size (LEfSe) analysis with linear discriminant analysis (LDA) score >3.5 at the age of 11 weeks. *Prevotellaceae_NK3B31_group*, *Rikenellaceae_RC9_gut_group*, and *Succinatimonas* were identified as differentially significant and biological feature taxa between groups (p<0.05).

The microbial composition in the cecum of RFCs raised in the control group showed that Firmicutes was the most dominant phylum, followed by Bacteroidetes, Proteobacteria, Epsilonbacteraeota, and Actinobacteria at the age of 4 weeks. The abundance of Firmicutes decreased with age following the increment of Bacteroidetes (Figure 4A). At the genus level, *Faecalibacterium* were predominant at the age of 4 weeks, followed by *Alistipes*, other unclassified genera, *Ruminococcus*, and *Helicobacter* in RFCs. The microbial composition changed with age and *Bacteroides* converted into the dominant genus from the age of 9 to 11 weeks. The supplementation of BA and SC did not alter microbial community structure with significance but increased the relative abundance of *Bernesiella*, *Lactobacillus*, and *Olsenella* when compared to RFCs fed with control diets (Figure 4B). Additionally, the supplementation significantly increased the cecal Firmicutes/Bacteroidetes (F/B) ratio at the age of 9 and 11 weeks (p<0.05; Figure 4C). Results of metagenomeSeq and STAMP analyses (Shown in Figure 5) on RFCs with growth benefits (at the age of 11 weeks) revealed the significantly increased abundance of *Bernesiella*, P*revotellaceae_NK3B31_group*, and *Butyrucimonas* (p<0.05) with remarkably decreased abundance of *Bacteroides*, *Rikenellaceae_RC9_gut_group*, and *Succinatimonas* (p<0.05). Among those genera, *Prevotellaceae_NK3B31_group*, *Rikenellaceae_RC9_gut_group*, and *Succinatimonas* were identified as differentially significant and biological feature taxa between groups by LEfSe with an LDA score of 3.5.

### Functional pathways promoted by feeding BA and SC

**Figure 6:**
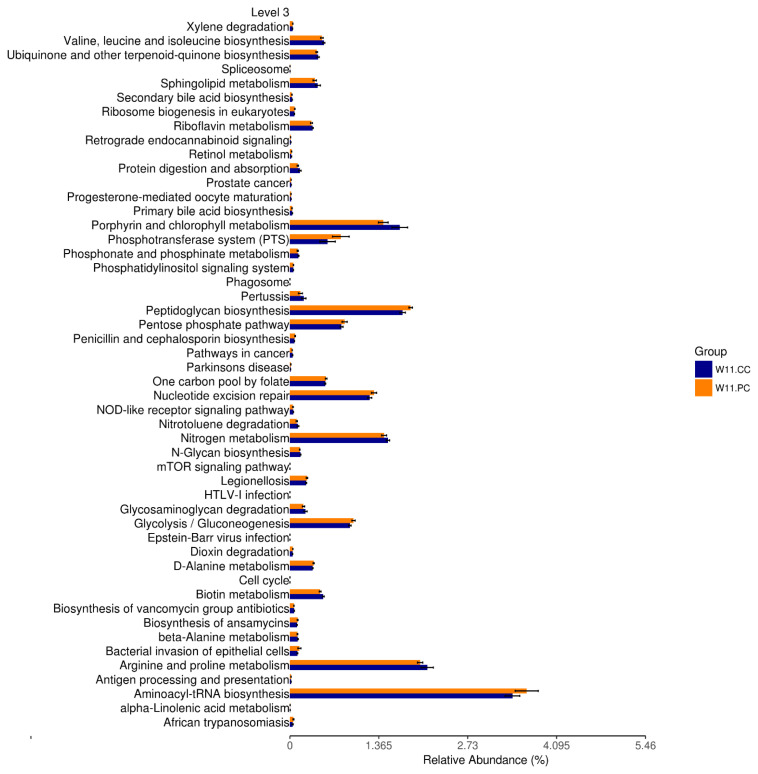
Functional prediction of KEGG pathways after by Tax4Fun. The third level of KEGG pathways was predicted between the control and experimental (BA+SC) groups at the age of 11 weeks (n = 6 per group). Pathways of peptidoglycan biosynthesis, nucleotide excision repair, glycolysis/gluconeogenesis, and aminoacyl transfer ribonucleic acid (tRNA) biosynthesis were significantly promoted. (*t*-test; p<0.05). KEGG, Kyoto encyclopedia of genes and genomes; BA, *Bacillus amyloliquefaciens*; SC, *Saccharomyces cerevisiae*.

In accordance with the growth benefits observed at the age of 11 weeks, functional predictions of KEGG pathways by Tax4Fun at the third level were conducted (Figure 6). The results demonstrated that the supplementation of BA and SC in feed significantly promoted the pathways of peptidoglycan biosynthesis, nucleotide excision repair, glycolysis/gluconeogenesis, and aminoacyl transfer ribonucleic acid (tRNA) biosynthesis (p<0.05).

## DISCUSSION

Several studies demonstrated that unitary addition of BA or SC in feed might increase BW, average daily gain, or average daily feed intake but compromise the FCR in broilers [16,17]. In the present study, the combination of BA Y2 and SC C1 improved BW and BWG of RFCs at the age of 9 and 11 weeks without compromise of FCR. The growth benefits were significant at the age of 11 weeks, indicating that this combination exerted synergistic effects that contributed to the observations [18]. Most studies have demonstrated that the mixed probiotics, such as *Lactobacillus* or *Bacillus* with SC, improved growth performance in broilers [19]. However, the growth benefits were not profound until the age of 11 weeks, disclosing that the combination of BA Y2 and SC C1 required long-term use to exhibit the significant growth benefits, especially for the breed with lengthy raising period, such as native chickens.

The measurements of intestinal villus heights, crypt depths, and V/C ratio at the level of small intestines are regularly applied for investigating the gut health and effects of various diet regimens in chickens [20]. Dietary feeding of probiotics has been demonstrated to improve the jejunal morphology in broilers [21]. The supplementation of BA Y2 and SC C1 in this study demonstrated the significant differences in the mucosal architecture, including increased villus heights, shortened crypt depths, and/or elevated V/C ratio in the jejunum and ileum. Notably, the significant effects were more comprehensive in small intestines at the age of 11 weeks, consistent with the observations in growth performance. Since the digestion and absorption of nutrients are dominated by the mucosa in the small intestines, the increments of villus heights provide a extended surface area capable of greater absorption. A higher V/C ratio represents a greater capacity of nutrient digestibility and absorption in chickens [22]. Furthermore, the lower V/C ratio is related to a low number of absorptive cells with a high number of secretory cells, leading to increased mucin production, compromised absorption of nutrients, and higher energy demands for maintaining intestinal function [23,24]. Although a significant difference in villus height was observed in the jejunum at the age of 9 weeks, the change did not impact growth performance. Improved mucosal architectures in both jejunum and ileum seemed essential to contribute to the significant growth benefits observed at the age of 11 weeks.

Several studies have shown that the number of microbial species in cecal microbiota increased with age in broiler and layer chickens. Firmicutes and Bacteroidetes dominated the bacterial phyla in the cecum and the abundance of Firmicutes decreased with age following the converse increment of Bacteroidetes [25,26]. Our observations in RFCs were consistent with those studies. Nevertheless, the long-term supplementation of BA and SC reversed the trend, significantly increasing the F/B ratio at the age of 9 and 11 weeks. A higher F/B ratio has been demonstrated to have a greater fermentation capacity of volatile fatty acids and more fat deposition, linking with the increased amount of energy and elevated BW in humans [27]. A higher F/B ratio represented improved energy uptake and growth promotion. The increase of the F/B ratio was associated with the desirable growth performance [26,28]. The significant increments of F/B ratio noted at the age of 9 weeks did not link with a significant performance improvement, indicating that the interactions of Firmicutes and Bacteroidetes are partially involved in the overall growth, or the index of F/B ratio was not a specific correlate with growth performance in RFCs. Further investigations of microbial species diversity and microbial shifts in gut microbiota at the detailed taxonomic level are necessary.

Animals with impaired gut health are characterized by the loss of species richness, and/or diversity and evenness in gut microbiota [29]. Up to date, the relationship between desirable growth performances, microbial species diversity, and microbial shifts in gut microbiota is not clear. Alpha diversity summarized the structure of the microbial community in the gut by species richness (the number of species present), species evenness (the number of individuals per species), or both. It is the first approach to evaluate differences between microbial environments. The supplementation of BA and SC in this study increased microbial diversity at the age of 9 and 11 weeks, particularly for species abundance at the age of 11 weeks. However, the differences were not significant between the control and experimental groups. The result indicated that the supplementation affected the abundance of taxa but did not alter the major community structure in the cecum. Beta diversity is a measure of similarity or dissimilarity between two microbial communities. Bray-Curtis dissimilarity was used to measure the shape (abundance of each taxon) and size (overall abundance per sample) of the communities. The supplementation of BA and SC in feed for 7 weeks promoted the differential separation of microbial composition structure from the un-supplemented. Afterward, the microbial communities between the two groups shared some degree of similarity. These analytic results of alpha and beta diversity demonstrated that the growth benefits promoted by supplementing BA and SC did not affect the majority of microbial community structure in the gut.

Several studies have been shown that the growth benefits of feeding DFM in broilers were correlated with the interaction with gut microbiota and the host. It was through modulating the composition of the intestinal microflora by the overgrowth of beneficial microbes and/or the decreasing population of pathogenic bacteria [8,30]. In the present study, the relative abundance of *Bernesiella*, *Lactobacillus*, and *Olsenella* in the group fed with BA and SC were found to be increased when the microbial composition targeting the top ten abundant taxa in the cecum was analyzed. Nevertheless, the increments were not significant. Therefore, several bioinformatics tools were applied to determine the statistical significance with biological consistency and effect relevance. The results of metagenomeSeq and STAMP showed that genera of *Bernesiella*, *Prevotellaceae_NK3B31_group*, and *Butyrucimonas* were significantly increased following remarkable decrements of *Bacteroides*, *Rikenellaceae_RC9_gut_group*, and *Succinatimonas* in RFCs with growth benefits. Among those genera, the increment of *Prevotellaceae_NK3B31_group* and decrements of *Rikenellaceae_RC9_gut_group* and *Succinatimonas* were determined as the significant biological feature by LEfSe. *Barnesiellais* is known as a member of the family *Porphyromonadaceae*, order *Bacteroidales*. It has been demonstrated to utilize fucosyllactose as the energy source, correlate with the amount of several immunoregulatory cells, exert anti-inflammatory effects in mice with dextran sulfate sodium-induced colitis, and prevent the colonization of vancomycin-resistant *Enterococcus* in the intestines [31–33]. *Prevotellaceae* was shown to be associated with the production of short-chain fatty acids (SCFAs) for modulating gut physiology and metabolism [34,35]. The *Prevotellaceae-NK3B31 group* are involved in the carbohydrate, amino acid, nucleotide metabolic, and lipid pathways [36]. A higher proportion of *Prevotellaceae_NK3B31_group* has been demonstrated to provide benefit to healthy pigs, alleviate diarrhea and promote growth performance in weaned piglets, and improve the average BW and intestinal morphology in piglets treated by lysozyme [37,38]. For *Butyricimonas*, it produces butyrate to either enhance intestinal barrier function and mucosal immunity or serve as the major energy source for the differentiation of colonocytes, thereby affecting the growth [39]. Increments in *Butyricimonas* abundance were shown to improve metabolic parameters in mice treated with metformin [40]. These results indicated that these featured taxa and their abundance may contribute to the major effects on growth performance in RFCs.

*Bacteroides* are considered to maintain a complex and beneficial relationship with the host. They ferment carbohydrates with other intestinal flora to produce a group of volatile fatty acids as an energy source for host utilization [41]. However, the amount of *Bacteroides* was negatively associated with abdominal fat and subcutaneous fat thickness in chickens [42]. The specific regulatory mechanism of *Rikenellaceae_RC9_gut_group* in the gut is unclear. One study showed that it was negatively linked with glucose metabolism parameters. The decrement of *Rikenellaceae_RC9_gut_group* may up-regulate the glucose metabolism, increasing the energy uptake by the host [43]. *Succinimonas* is recognized as a novel member of the family *Succinivibrionaceae* of the class *Gammaproteobacteria* [44]. It was isolated from human feces and shown to ferment carbohydrates to succinate and acetate [45]. Nevertheless, few studies addressed the role of *Succinimonas* in growth performance. Based on evidence mentioned above, it is speculated that the increments of *Bernesiella*, *Prevotellaceae_NK3B31_group*, and *Butyrucimonas* might associate with more energy uptake in the host, modulations of gut physiology and metabolism, and enhancements of intestinal barrier function and mucosal immunity, consequently contributing to the growth benefits in RFCs. Although some research highlighted the role of *Bacteroides* in growth promotion, the relationship was not established in this study. It indicated that the growth benefits from supplementing BA and SC were not through the interactions with this genus of bacterium and RFCs. Human studies further demonstrated that the microbial shift from *Bacteroides* to *Prevotellaceae_NK3B31_group* in the colon revealed the metabolic changes of carbohydrate, amino acid, nucleotide, and lipid [36].

The intestinal microbiota has been demonstrated to participate in several metabolic pathways, including amino acid synthesis and lipid metabolism [46]. Predictions of functional pathways in the present study demonstrated significant promotions of peptidoglycan biosynthesis, nucleotide excision repair, glycolysis/gluconeogenesis, and aminoacyl-tRNA biosynthesis by supplementing BA and SC. These results showed the participation of cecal featured taxa in metabolic functions. The peptidoglycan as a macromolecular component of the bacterial cell wall maintains the shape and integrity of the cell and forms a thick layer in the cell wall of lactic acid bacteria (LAB) [47]. LAB strains, *Lactobacillus* in particular, are commercially used as probiotics with health or growth-promoting properties [48]. Increased biosynthesis of peptidoglycan may enhance the abundance of LAB populations in the gut, partially promoting the growth benefits. A recent study has shown the positive role of *Prevotellaceae_NK3B31_group* in amino acid, carbohydrate, lipid, and nucleotide metabolic pathways, influencing the concentration of acetic acid, propionic acid, and total SCFAs in the intestinal digesta [36]. The higher abundance of this genus may promote the higher predicted carbohydrate and nucleotide metabolic pathways. Another study demonstrated a negative correlation between the abundance of *Rikenellaceae_RC9_gut_group* and glucose metabolism [43], indicating that its decrement contributed to higher predicted glucose metabolic function. The aminoacyl tRNA is required for protein biosynthesis [49]. It was found to involve the peptidoglycan cross-linking pathway, antibiotic resistance, antibiotic synthesis, and membrane phospholipid modification pathways [50]. Nucleotide excision repair is the mechanism that protects cells against genomic damage and its disruption can result in tumorigenesis and accelerate aging [51]. Deficiencies in nucleotide excision repair were also associated with the inferior function of cellular metabolism [52]. Therefore, significant promotion on this pathway was beneficial for the maintenance of mucosal architectures in the intestine. However, further studies are required to examine the speculation. According to these pieces of evidence, the microbial shifts in response to the supplementation of BA and SC may play the role in the metabolic and physiological regulations, subsequently contributing to the growth benefits in RFCs.

## CONCLUSION

The supplementation of BA Y2 and SC C1 in feed exerts beneficial effects on growth performance and gut mucosa improvement in RFCs. The supplementation did not disturb the major microbial community structure but promote the microbial shifts characterized by the increments of *Bernesiella*, *Prevotellaceae_NK3B31_group*, and *Butyrucimonas*, following the decrements of *Bacteroides*, *Rikenellaceae_RC9_gut_group*, and *Succinatimonas*. Evidence showed that these featured taxa and their abundance may contribute to benefits on growth performance. Modulated cecal microbiota was demonstrated to play a role in chicken metabolic and physiological regulations through functional predictions.

## Figures and Tables

**Table 1:** Ingredients and composition of the control diets

Items	Starter diet (1 to 21 days)	Grower diet (22 to 42 days)	Finisher diet (43 to 77 days)
Ingredients (g/kg)	-------------------------------------------------------------------------g/kg------------------------------------------------------------------------------		
Corn, yellow	555	586	601
Soybean meal	295	260	230
Full fat soybean meal	50	75	100
Fish meal (CP-65%)	50	25	12.5
Soybean oil	18	21	24
Monocalcium phosphate	13	14	14
Calcium carbonate	11.5	11	10.5
DL-methionine	2	1.6	1.5
NaCl	2.5	3	3.5
Choline-Cl	1	1	1
Vitamin premix^1)^	1	1	1
Mineral premix^2)^	1	1	1
Total	1,000	1,000	1,000
Calculated nutrient value			
ME (kcal/kg)	3,025	3,075	3,125
Crude protein (%)	22	20	19
Calcium (%)	1	0.9	0.85
Total phosphorus (%)	0.64	0.6	0.56
Available phosphorus (%)	0.5	0.45	0.2
Lysine (%)	1.29	1.13	1.04
Methionine (%)	0.6	0.51	0.48
Methionine+Cystein (%)	0.95	0.85	0.8
Threonine (%)	0.86	0.78	0.73
Cl (%)	0.21	0.23	0.25
Na (%)	0.17	0.17	0.17
K (%)	0.89	0.85	0.83

ME, metabolizable energy.

1) Supplied per kg of diet: Vit A 15,000 IU; Vit. D_3_ 3,000 IU; Vit. E 30 mg; Vit. K_3_ 4 mg; riboflavin 8 mg; pyridoxine 5 mg; Vit. B_12_ 25 μg; Ca-pantothenate 19 mg; niacin 50 mg; folic acid 1.5 mg; biotin 60 μg.

2) Supplied per kg of diet: Co(CoCO_3_) 0.255 mg; Cu(CuSO_4_·5H_2_O) 10.8 mg; Fe(FeSO_4_·H_2_O) 90 mg; Zn(ZnO) 68.4 mg; Mn(MnSO4·H2O) 90 mg; Se (Na_2_SeO_3_) 0.18 mg.

**Table 2:** The effects of dietary supplementations on growth performance of red-feathered native chickens (RFCs) at the age of 9 and 11 weeks

Parameters	Group		p-value

	Control	Experimental	
9 weeks			
BW (kg/bird)	1.82±0.13	1.87±0.17	0.084
BWG (kg/bird)	1.77±0.13	1.82±0.17	0.085
FCR	2.32	2.25	
11 weeks			
BW (kg/bird)	2.35±0.14	2.46±0.19^*^	0.002
BWG (kg/bird)	2.30±0.14	2.41±0.19^*^	0.002
FCR	2.45	2.34	

BW, body weight; BWG, body weight gain; FCR, feed conversion rate.

BW and BWG values were reported as mean±standard deviation, n = 50 per group.

FCR was measured by total amount of feed consumption by the total weight gain from all RFCs in the group.

* Means are significantly different when compared to the control (p<0.05).

**Table 3:** Differences in jejunal and ileal villus heights, crypt depths, and V/C ratios of RFCs between control and experimental (BA+SC) groups at different ages

Parameters	Jejunum		p-value		Ileum	p-value
	
	Control (Mean±SD)	Experimental (Mean±SD)		Control (Mean±SD)	Experimental (Mean±SD)	
9 weeks						
Villus height (μm)	1,082.5±69.07	1,287.49±160.32^*^	0.017	953.3±211.6	1,126.7±190.53	0.167
Crypt depth (μm)	285.38±86.05	265.06±24.35	0.590	280.74±84.94	234.65±26.59	0.233
Villus height/crypt depth	4.26±1.15	5.11±0.89	0.183	3.91±1.41	5.05±1.22	0.164
11 weeks						
Villus height (μm)	1,194.63±136.26	1,256.56±127.55	0.435	831.24±94.49	971.47±76.86^*^	0.018
Crypt depth (μm)	263.46±15.87	228.91±21.46^*^	0.010	204.65±26.40	161.83±22.7^*^	0.013
Villus height/crypt depth	4.77±0.57	5.74±0.8^*^	0.035	4.39±0.69	6.39±0.62^*^	0.0004

All values were reported as mean±standard deviation (SD), n = 6 per group.

* Means are significantly different when compared to the control (p<0.05).
